# Inter-Rater Reliability of the Progressive Pain Check Method for Standardized Pain Assessment in Lipedema

**DOI:** 10.3390/life16091471

**Published:** 2026-09-02

**Authors:** Laura Patton, Lorenzo Ricolfi, Heba Safwat Mhmoued Abdo Elhadidy, Valeria Reverdito, Antonino Russotto, Gianfranco Politano, Maria Michela Gianino

**Affiliations:** 1 Endocrinology and Lymphology Clinic, 38096 Vallelaghi, Italy; lauraptt77@gmail.com (L.P.); lorenzoricolfi@hotmail.it (L.R.); 2 Department of Public Health Sciences and Paediatrics, University of Turin, 10126 Torino, Italy; antonino.russotto@unito.it (A.R.); mariola.gianino@unito.it (M.M.G.); 3 Human Project Studio, 10138 Torino, Italy; valeria.reverdito@gmail.com; 4 Department of Control and Computer Engineering, Politecnico di Torino, 10129 Torino, Italy; gianfranco.politano@polito.it

**Keywords:** lipedema, pain assessment, allodynia, inter-rater reliability, clinical, examination

## Abstract

Background: Lipedema is a chronic progressive condition affecting approximately 10% of women, characterized by disproportionate subcutaneous adipose tissue accumulation and pain in the lower extremities. Standardized assessment methods for pain evaluation in lipedema remain limited. The Progressive Pain Check (PPC) method represents a potentially valuable tool for standardized pain assessment in lipedema patients. Objective: To evaluate the inter-rater reliability of the PPC method for clinical assessment of evoked pain in lipedema patients. Methods: Two independent assessors performed PPC evaluations. Inter-rater reliability was assessed using the Intraclass Correlation Coefficient [ICC(2,1), two-way random-effects, absolute agreement, single-measurement] with 95% confidence intervals (CI). Results: This study included 429 women diagnosed with lipedema (mean age 41.2 ± 11.5 years, mean disease duration 27.2 ± 12.6 years). Patient characteristics revealed mean BMI of 29.5 ± 6.9 kg/m^2^, predominantly Type III lipedema presentation, and disease onset during adolescence (mean age 14 ± 9.3 years). The PPC method demonstrated excellent inter-rater reliability between two independent assessors across all examined anatomical districts. Conclusions: The PPC method shows promise as a simple, repeatable, and standardized clinical tool for evoked pain assessment in lipedema patients. The demonstrated inter-rater reliability supports its potential utility in clinical practice. Further research is warranted to evaluate its intra-rater reliability, concurrent validity, and responsiveness in monitoring treatment effects.

## 1. Introduction

Lipedema is a chronic, progressive disorder characterized by symmetrical, disproportionate fat accumulation in the lower limbs and, less frequently, the upper arms, with epidemiological studies suggesting an estimated prevalence ranging from 6–8% to 15–19% in different female populations [1]. Despite its long-standing recognition, lipedema remains underdiagnosed and poorly integrated into healthcare systems globally. Diagnostic delays averaging 15 years persist due to overlapping features with obesity and lymphedema, leaving patients vulnerable to progressive physical disability and inadequate therapeutic interventions [2]. The Allen-Hines diagnostic criteria remain foundational but struggle to differentiate lipedema from comorbid conditions [3]. Key distinguishing features include fat distribution, characterized by symmetrical limb involvement that spares the feet, unlike the generalized pattern of obesity or the distal-to-proximal progression of lymphedema; pain characteristics, featuring spontaneous tenderness and pressure-induced pain that differ from obesity-related discomfort or the sensation of heaviness typical of lymphedema; and vascular signs, notably a pronounced bruising tendency [4].

This overlap leads to frequent misdiagnosis, as a high rate of patients initially receive incorrect labels like “morbid obesity” or “idiopathic edema,” delaying appropriate care [4,5].

Pain represents the most debilitating symptom, manifesting as allodynia, mechanical hyperalgesia, and spontaneous tenderness [6]. These phenotypes suggest mixed nociceptive and neuropathic mechanisms [7,8]. Recent studies have further investigated the relationship between tissue characteristics, stiffness, and pain in lipedema, emphasizing the need for structured pain evaluation [9]. An accurate assessment of pain is crucial for improving patient outcomes in lipedema, as it directly influences therapeutic decisions and the effectiveness of interventions. Several scales have been proposed, such as the Visual Analog Scale (VAS) and Numeric Rating Scale (NRS), which are designed for acute or postoperative pain and fail to capture lipedema’s chronic mixed pain profile (nociceptive + neuropathic); the Short Form Health Survey 36 (SF-36), which measures global impairment but lacks disease-specific metrics; and Pressure Pain Threshold (PPT) Testing, which is objective but requires specialized equipment unavailable in most clinics [10]. None of these scales have been specifically tailored to capture lipedema-specific pain dimensions, as they were validated for musculoskeletal or neuropathic conditions [11]. This highlights the necessity for disease-specific tools that can provide more precise and reliable evaluations. A validated pain scale specifically designed for lipedema would help clinicians make better therapeutic decisions and improve patient outcomes. Furthermore, the lack of consensus on the most appropriate methodology for pain assessment hinders the comparability of studies and the development of evidence-based therapeutic strategies.

Therefore, this study aims to address these gaps by assessing the inter-rater reliability of the Progressive Pain Check (PPC) method, which was developed based on our preliminary research indicating its potential to capture the unique pain characteristics associated with lipedema [12]. The PPC offers a more tailored approach to pain assessment, addressing the limitations of existing scales. The primary objective is to evaluate the inter-rater reliability of the PPC method between two independent assessors.

## 2. Materials and Methods

### 2.1. Study Design and Participants

This was an observational retrospective study, conducted between the 16 August 2023 and 31 January 2025 across private specialist clinics. The study aimed to evaluate the inter-rater reliability of the PPC method using retrospectively collected clinical records. Written informed consent for the use of clinical data was obtained from all the recruited patients.

Inclusion criteria comprised female patients with a clinical diagnosis of lipedema, ability to provide informed consent, and stable clinical condition. Exclusion criteria included stage IV lipedema, concomitant lymphedema, active infections or open wounds in the assessment areas, and inability to undergo physical examination. The diagnosis of lipedema was established according to standard international clinical criteria: bilateral and symmetrical fat distribution in the lower and/or upper limbs with characteristic sparing of hands and feet, presence of subcutaneous nodules, negative Stemmer’s sign, and pressure-induced pain, in addition to other symptoms characteristic of the disease [1]. Clinical examination included comprehensive evaluation of the entire body, with assessment of lipedema tissue distribution, limb conformation, subcutaneous tissue characteristics, and presence of associated signs such as edema, vascular changes, and endocrinological manifestations. Each participant underwent evaluation using the PPC method, a systematic bedside protocol designed to assess pressure-induced pain and mechanical allodynia characteristic of lipedema tissue across standardized anatomical landmarks. The PPC evaluation encompasses 11 anatomical points across lower and upper body regions. For the lower body assessment, there are eight points:

1. medial lower third of the leg (approximately 5 cm above the medial malleolus);
2. posterior middle third of the leg (immediately below the gastrocnemius muscle);
3. medial upper third of the leg (medial to the tibial tuberosity prominence);
4. medial lower third of the thigh (assessed by applying pressure to tissue in the popliteal space);
5. medial upper third of the thigh (approximately 5 cm below the inguinal crease);
6. lateral upper third of the thigh (immediately below the trochanter);
7. lateral lower third of the thigh (approximately 5 cm above and lateral to the patella);
8. lower abdomen (below an imaginary line between the upper pelvic bones and 3 fingers below the navel);

For the upper body assessment, there are three points:

1. lateral edge of the tissue covering the teres major muscle;
2. arm;
3. forearm.

Standardization of the PPC procedure involved lifting a full-thickness subcutaneous adipose tissue fold between the thumb and index finger, applying a firm, sustained compression for approximately 1–2 s. For the medial lower third of the thigh, where tissue fold lifting is anatomically restricted, pressure was applied between the four fingers and the thenar eminence/palm of the hand. Assessments were performed systematically in a predefined sequence, starting distally on the lower limb and proceeding proximally, followed by the abdomen, back, and upper extremities, evaluated on the patient’s right side in the supine and standing positions.

At each anatomical point, evoked pain intensity was reported by the patient using a 5-point Verbal Rating Scale (VRS): 0 = no pain, 1 = mild pain, 2 = moderate pain, 3 = severe pain, 4 = very severe pain. Three summary scores were calculated: the Lower Body Pain Score (LBPS), defined as the sum of the eight lower-body points (range: 0–32); the Upper Body Pain Score (UBPS), calculated as the sum of the three upper-body points (range: 0–12); and the Total Body Pain Score or Ricolfi–Patton Score (RP Score), representing the sum of all 11 evaluated points (range: 0–44) [12].

The PPC evaluation was performed independently by two trained specialist medical doctors (one lymphologist and one endocrinologist) experienced in lipedema management. Both evaluators had previously calibrated their examination techniques and landmark identification. To ensure blinding and independent rating, the assessors conducted their physical examinations during separate clinical visits, and both were blinded to each other’s recorded scores.

### 2.2. Statistical Analysis

Continuous variables were summarized as mean ± standard deviation (SD) or median and interquartile range (IQR), while categorical variables were reported as absolute numbers and percentages.

Inter-rater agreement for the PPC scores was assessed using the Intraclass Correlation Coefficient (ICC) with a two-way random-effects model, absolute-agreement definition, and single-measurement unit [ICC(2,1)]. This model was selected because each participant was assessed by two raters, and the aim was to quantify absolute agreement between individual assessments rather than merely consistency or rank-order association. ICC estimates and 95% confidence intervals (CIs) were calculated separately for the lower-body score, upper-body score, and total PPC score. ICC values were interpreted according to standard benchmarks: poor (<0.50), moderate (0.50–0.69), good (0.70–0.90), or excellent (>0.90) reliability [13]. Descriptive statistics are reported as mean ± SD for each rater, together with the mean between-rater difference. In addition, inter-rater concordance across individual anatomical sites and lipedema phenotypes was evaluated.

All statistical tests were two-tailed, and *p*-values < 0.05 were considered statistically significant. Analyses were performed using R version 4.3.1 (R Core Team, Vienna, Austria, 2023).

### 2.3. Ethics

This study was conducted in accordance with the Declaration of Helsinki (1964) and its subsequent amendments, as well as Good Clinical Practice (GCP) guidelines. The study protocol received approval from the “Comitato Etico Territoriale della Provincia Autonoma di Trento per le sperimentazioni cliniche” (CET-PAT) (approval number: A909). All participants provided written informed consent prior to enrollment after receiving comprehensive information about study objectives, procedures, potential risks and benefits, and their right to withdraw at any time without affecting their ongoing clinical care.

The informed consent process was conducted in a private setting, allowing participants adequate time to ask questions and consider participation. Patient confidentiality and data protection were maintained throughout the study in accordance with applicable data protection regulations. All data were anonymized, and personal identifying information was stored separately from study data in secure, access-controlled databases. Only authorized research personnel had access to study data, and all electronic data were password-protected and encrypted during storage and transmission.

No artificial intelligence (AI) was used in the study and writing of the article.

## 3. Results

A total of 429 patients with clinically diagnosed lipedema were enrolled in this study. The mean age of participants was 41.2 years (SD = 11.5), with a median age of 41 years (IQR: 33–49 years). Age distribution approximated normal distribution with the majority of participants falling within the fourth and fifth decades of life. Regarding disease onset, participants reported a mean age at onset was 14 years (SD = 9.3), while the median age at subjective recognition of the first symptoms was 12 years (IQR = 10–14 years). The median duration of disease at study enrollment was 27 years (IQR = 18–36 years). Body mass index (BMI) analysis revealed a median of 28.1 kg/m^2^ (IQR = 23.73–33.31 kg/m^2^). The detailed description of the sample is shown in Table 1.

Inter-rater reliability analysis demonstrated excellent absolute agreement between the two independent assessors across all summary PPC scores. LBPS: mean score was 24.76 ± 5.81 for Assessor 1 and 24.79 ± 5.83 for Assessor 2, with a mean between-rater difference of +0.03. The ICC(2,1) was 0.9908 (95% CI: 0.9889–0.9924; F(428, 428) = 217.29, *p* < 0.001); UBPS: mean score was 4.32 ± 3.40 for Assessor 1 and 4.30 ± 3.25 for Assessor 2, with a mean between-rater difference of −0.03. The ICC(2,1) was 0.9745 (95% CI: 0.9692–0.9788; F(428, 428) = 77.24, *p* < 0.001); RP Score: mean total score was 29.08 ± 8.11 for Assessor 1 and 29.08 ± 7.97 for Assessor 2, with a mean difference of 0.00. The ICC(2,1) was 0.9904 (95% CI: 0.9885–0.9921; F(428, 428) = 207.82, *p* < 0.001).

**Figure 1:**
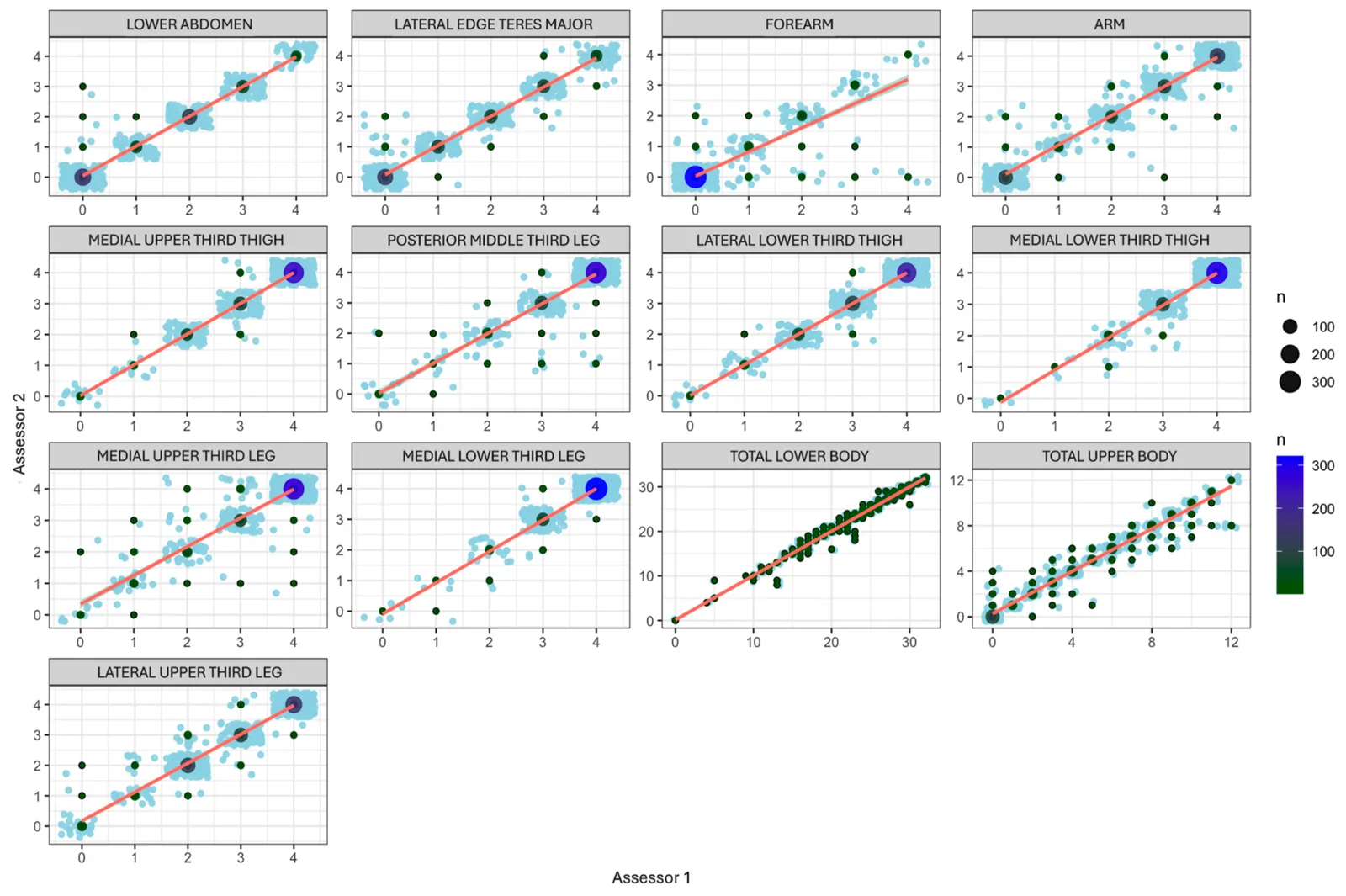
Inter-rater reliability and agreement between Assessor 1 and Assessor 2 across the 11 individual anatomical points and composite summary scores (Total Lower Body, Total Upper Body). (*n* = 429).

As illustrated in Figure 1, inter-rater consistency remained consistently high across all 11 individual anatomical points defined in the PPC protocol. The highest levels of concordance were observed in the lower extremities and trunk, including the medial and lateral lower third of the thigh, medial upper third of the thigh, and lower abdomen (r ≥ 0.98), followed by the arm and lateral edge of the teres major (r = 0.97–0.98). The forearm district exhibited slightly lower but still robust agreement (r = 0.86). All individual site comparisons reached statistical significance (*p* < 0.001).

**Figure 2:**
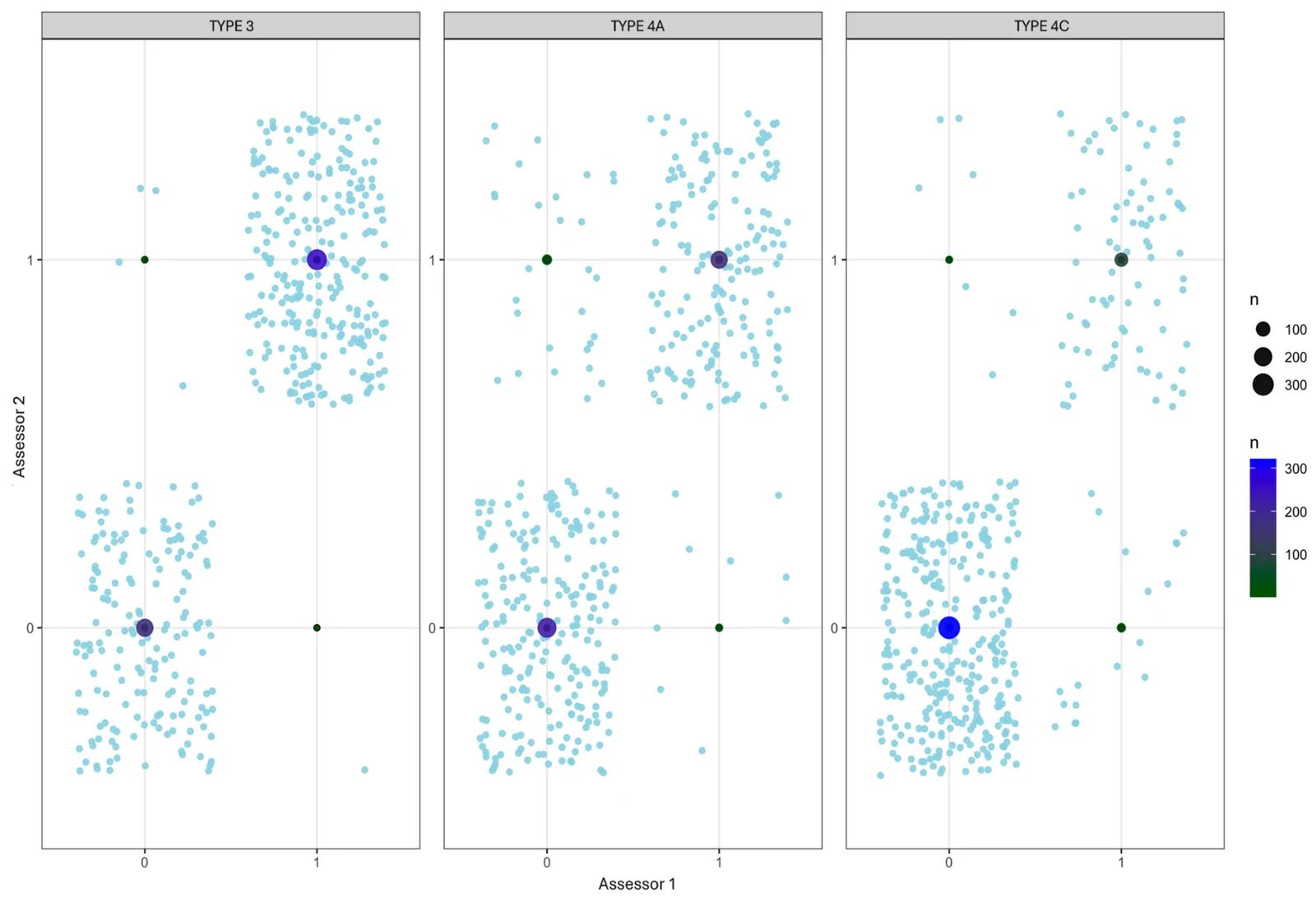
Inter-rater concordance between Assessor 1 and Assessor 2 for the clinical classification of lipedema phenotypes: Type 3, Type 4a, and Type 4c. (*n* = 429).

As shown in Figure 2, inter-rater agreement for the identification of affected anatomical subtypes yielded concordant results. The classification agreement for Type 3 lipedema presentation showed a correlation coefficient of 0.98 between the two independent assessors (*p* < 0.001). For upper extremity phenotypes, Type 4a involvement (extension to the elbow) demonstrated a coefficient of 0.82, while Type 4c involvement (extension to the wrist) showed a coefficient of 0.83 (both *p* < 0.001).

Secondary analyses evaluated the relationship between PPC evoked pain scores and clinical phenotypic distribution. For lower-body assessments, PPC evoked scores showed consistent association with the clinical extent of lower limb disease (Assessor 1: r = 0.476, *p* < 0.001; Assessor 2: r = 0.474, *p* < 0.001). For upper extremity assessments, PPC scores were strongly correlated with upper limb phenotypic involvement (Assessor 1: r = 0.848, *p* < 0.001; Assessor 2: r = 0.934, *p* < 0.001).

## 4. Discussion

The main objective of this study was to evaluate the inter-rater reliability of the PPC method as a standardized clinical tool for evoked pain assessment in lipedema, addressing the limitations of existing generic pain scales that fail to capture the unique pain characteristics of this condition [7,11]. By addressing these gaps, this study seeks to contribute to the development of standardized clinical assessment methods for lipedema, ultimately improving patient care and management.

Our results demonstrate that the PPC exhibits excellent inter-rater reliability across all anatomical regions examined, with high ICC and minimal between-rater differences for lower-body, upper-body, and total pain scores. These findings were consistent across different lipedema types, with type 3 achieving the highest consistency, and types 4a and 4c also demonstrating robust agreement. Secondary analyses confirmed significant associations between PPC evoked scores and the anatomical distribution and clinical staging of lipedema.

The high inter-rater reliability of the PPC across both lower and upper limb regions is notable. This consistency is particularly relevant given the challenges in lipedema diagnosis, which is frequently confused with obesity and lymphedema [4,14]. It is essential to recognize that lipedema, obesity, and lymphedema frequently coexist, as evidenced by the BMI distribution in our cohort. In these cases, obesity-related systemic inflammation and mechanical load may exacerbate tissue tenderness [4]. The slightly lower inter-rater agreement in the forearm region may be attributed to the more variable phenotypic involvement of the upper extremities in lipedema, consistent with clinical observations and previous literature [15,16]. The outstanding agreement for type 3 lipedema supports the PPC’s reproducibility across widespread lower extremity involvement, while the solid consistency for types 4a and 4c suggests that the tool remains reliable even when assessment complexity increases due to upper limb extension.

The moderate correlation between PPC scores and clinical disease severity in the lower extremities suggests that while pressure-evoked pain is a key feature of lipedema, it is complementary to other essential diagnostic criteria, as described before [1,17]. Furthermore, it is important to consider that evoked pain may be influenced by comorbid conditions sharing chronic low-grade inflammation, including fibromyalgia, chronic venous disease, metabolic dysregulation, and peripheral neuropathies [18]. While the PPC specifically targets the mechanical hypersensitivity and allodynia characteristic of lipedema tissue, findings must be interpreted within a comprehensive clinical framework.

The PPC’s ability to assess allodynia, mechanical hyperalgesia, and spontaneous tenderness makes it a valuable addition to both clinical practice and research, where standardized pain assessment is crucial for monitoring disease progression and evaluating therapeutic interventions. The tool’s simplicity and ease of use make it accessible for a wide range of healthcare professionals, including physiotherapists and nutritionists, who play a key role in the multidisciplinary management of lipedema [19,20]. Healthcare professionals, in particular, can benefit from the PPC as it allows for standardized monitoring of pain over time, facilitating the evaluation of treatment efficacy and supporting early suspicion and referral for specialized care. This is especially important given the long diagnostic delays and the significant impact of lipedema on quality of life [17,21]. However, it is essential to emphasize that the PPC is designed to complement, not replace, comprehensive physical examination and medical history, which remain foundational according to current clinical guidelines [1,17].

This study has several limitations that should be considered when interpreting the results. First, the observational retrospective design may introduce selection bias, as participants were recruited from specialized outpatient clinics with experience in lipedema management, potentially limiting generalizability to broader healthcare settings. Second, given the long disease duration in our cohort, many patients may have undergone prior pharmacological or conservative treatments (e.g., anti-inflammatory drugs or manual lymphatic drainage) before our evaluation. While this reflects common clinical reality, it may influence the baseline pain scores. Third, although inter-rater reliability was excellent, the study did not assess intra-rater reliability, which would provide additional information on measurement consistency over time by the same examiner. It is also important to consider that while the two independent assessors were blinded to each other’s scores, they are also authors of the study. Therefore, validation by external healthcare professionals is required to ensure the generalizability of the PPC. Furthermore, no comparison was conducted with existing pain scales: establishing concurrent validity with these established methods remains a necessary step for future research. Finally, while the PPC demonstrated robust reliability, its responsiveness to therapeutic interventions and sensitivity to clinical change over time were not evaluated and require further studies.

## 5. Conclusions

In conclusion, the PPC method demonstrates excellent inter-rater reliability and agreement between independent assessors for the structured evaluation of evoked pain in patients with lipedema. It represents a simple, repeatable, and non-invasive clinical protocol to map tissue tenderness and mechanical allodynia across standardized anatomical landmarks. While these findings support its consistency during physical examination, the PPC should be considered a complementary instrument supporting clinical judgment. Future prospective studies should focus on establishing intra-rater reliability, determining diagnostic cut-off values for the composite scores (LBPS, UBPS, and RP Score), assessing concurrent validity against instrumented algometry, and evaluating its responsiveness to conservative and surgical therapeutic interventions.

## Figures and Tables

**Table 1:** Demographic and clinical characteristics of enrolled patients.

Parameters	
Age (years)—mean (sd)	41.2 (11.5)
Age of onset (years)—mean (sd)	14 (9.3)
Duration of disease (years)—mean (sd)	27.2 (12.6)
Body mass index (kg/m^2^)—mean (sd)	29.5 (6.9)
Body weight (kg)—mean (sd)	78.5 (18.1)
Waist circumference (cm)—mean (sd)	93.8 (14.6)
Hip circumference (cm)—mean (sd)	115.3 (12.4)
Type I lipedema—*n* (%)	5 (1.2%)
Type II lipedema—*n* (%)	22 (5.1%)
Type III lipedema—*n* (%)	398 (92.8%)
Type IV lipedema—*n* (%)	304 (70.9%)
Type V lipedema—*n* (%)	4 (0.9%)
Stage 1 lipedema—*n* (%)	176 (41.1%)
Stage 2 lipedema—*n* (%)	152 (35.4%)
Stage 3 lipedema—*n* (%)	101 (23.5%)

## Data Availability

The original contributions presented in this study are included in the article. Further inquiries can be directed to the corresponding author.

## Abbreviations

The following abbreviations are used in this manuscript:

AI	Artificial Intelligence
BMI	Body Mass Index
CET-PAT	Comitato Etico Territoriale della Provincia Autonoma di Trento
CI	Confidence Interval
GCP	Good Clinical Practice
ICC	Intraclass Correlation Coefficient
IQR	Interquartile Range
LBPS	Lower Body Pain Score
NRS	Numeric Rating Scale
PPC	Progressive Pain Check
PPT	Pressure Pain Threshold
QST	Quantitative Sensory Testing
RP	Ricolfi-Patton
SD	Standard Deviation
SF-36	Short Form Health Survey 36
UBPS	Upper Body Pain Score
VAS	Visual Analog Scale
VRS	Verbal Rating Scale
